# Supplementary material for: Long-Term Care and Follow-Up in Laryngeal Cancer Patients: A Multicenter Retrospective Analysis
Source: J Pers Med. 2023 May 31;13(6):927. doi: 10.3390/jpm13060927 (PMC10302671; doi:10.3390/jpm13060927)
Supplement: Supplementary file 1 [file jpm-13-00927-s001.zip › jpm-2423650-supplementary.pdf]

Table S1. Treatment modality for SPTs

Patient number	Localization	Treatment modality	Outcome
1	Oral cavity	RT (60Gy)	NED
2	Kidney	Partial nephrectomy	NED
3	Lung (small cell lung cancer)	No therapy	DoD
4	Colon	Hemicolectomy, chemotherapy (Mayo protocol, XELIRI protocol, FOLFIRI protocol)	DoD
5	Oral cavity	Chemotherapy (CDDP+5-FU)	DoD
6	Lung	No therapy	DoD
7	Rectum	No therapy	NED
8	Prostate	RT (74 Gy)	NED
9	Larynx	Total laryngectomy	DoD
10	Colon	Rectosigmoid resection	NED
11	Brain	Surgery	NED
12	Oesophagus, thoracic part	No therapy	DoD
13	Oesophagus, cervical part	Pharyngoesophagectomia, RT (64 Gy)	DoD
14	Bile duct	No therapy	DoD
15	Lung	No therapy	DoD
16	Prostate	Radical prostatectomy and pelvic limphadenectomy, RT 68 (Gy)	NED
17	Oropharynx, cervical oesophagus, neck metastases	No therapy	DoD
18	Lung	Lobectomy	DoD
19	Lung	Chemotherapy (Cisplatin and Gemcitabine), RT (60Gy)	DoD
20	Oropharynx	Surgery, RT (60 Gy)	DoC
21	Rectum	Decision for therapy in progress	NED
22	Pancreas	No therapy	DoD
23	Colon	No therapy	NED
24	Squamous cell carcinoma of the skin, parietal region	RT (56 Gy)	DoC
25	Lung	Chemotherapy (Cisplatin and Gemcitabine)	DoD
26	Merkel cell cancer of elbow with metastases in axilla	Surgery and RT (54 Gy)	NED
27	Lung	RT (66 Gy)	NED
28	Oesophagus, cervical part	No therapy	DoD
29	Larynx, melanoma dorsi	RT (70 Gy) for larynx, surgical excision for melanoma	NED
30	Oropharynx, lung	chemoradiotherapy (CDDP) for oropharynx, lobectomy and chemotherapy (CDDP/5-FU) for lung	NED

31	Lung	RT (60 Gy)	DoD
32	Oesophagus, thoracic part	No therapy	DoD
33	Oesophagus, cervical part	No therapy	DoD
34	Pleural mesothelioma	Chemotherapy (CCDP)	DoD
35	Lung	Chemotherapy (Carboplatinum and Pemetreksed)	DoD
36	Rectum	Resection of rectum	DoD
37	Prostate	TURP	NED
38	Larynx	Total laryngectomy	DoC
39	Prostate	Radical prostatectomy and pelvic limphadenectomy	NED
40	Oropharynx	Partial pharyngectomy, RT (60 Gy)	NED
41	Melanoma dorsi	Surgical excision	NED
42	Prostate, neopharynx, lung	Radical prostatectomy and Eligard, partial pharyngectomy and RT (30 Gy) for oropharynx, RT (30Gy) for lung cancer	DoD
43	Urinary bladder	Radical cistectomy	NED
44	Non-Hodgkin lymphoma	Chemotherapy (CHOP protocol) + methotrexate	DoC
45	Urinary bladder, lung	TUR, chemotherapy (Gemcitabine + carboplatinum)	DoD
46	Lung	Lobectomy	DoD
47	Prostate with metastases in bones	Eligard + Docetaxelum	NED
48	Stomach	Gastrectomia distalis cum anastomosis gastrojejunalis	NED
49	Pancreas	No therapy	DoD
50	Urinary bladder	TUR	DoC
51	Acute myloid leucemia	Polychemotherapy	NED
52	Non-Hodgkin lymphoma	Polychemotherapy	DoD
53	Breast	Mastectomy	NED
54	Larynx	Total laryngectomy	DoD
55	Pancreas	Whipple operation + chemotherapy (Gemcitabine)	NED
56	Cecum	Chemotherapy (RP protocol)	NED
57	Rectum	Resection of rectum, RT (60 Gy)	DoD
58	Lung	Chemotherapy (Carboplatinum + Gemcitabine)	DoD
59	Lung	Palliative RT (20 Gy)	DoD
60	Thyroid gland	Total thyroidectomy	NED
Abbreviations: RT= radiotherapy; NED= no evidence of disease; DoD= died of disease; DoC= died of other causes			
Data is available for 60 (65,9%) of the 91 patients			

Table S2. Treatment modality for tumor recurrence

Patient number	Localization	Treatment modality	Outcome
1	Neck	Neck dissection	NED
2	Larynx	Total laryngectomy, thyroid lobectomy, neck dissection	NED
3	Trachea	No therapy	DoD
4	Lung	Chemotherapy (CDDP/5FU)	DoD
5	Tracheostoma, neck	Neck dissection, palliative RT (30-45 Gy)	DoD
6	Tracheostoma	Palliative RT (30-45 Gy)	DoD
7	Neck	Neck dissection, palliative RT (30-45 Gy)	DoD
8	Lung	Palliative RT (30-45 Gy)	DoD
9	Lung	No therapy	DoD
10	Skin infiltration	Surgery, Cetuximab	DoD
11	Lung	Palliative RT (30-45 Gy)	DoD
12	Neck, lung	Neck dissection, chemotherapy (CDDP/5FU)	NED
13	Esophagus	Palliative RT (30-45 Gy)	DoD
14	Esophagus	No therapy	DoD
15	Neck	Neck dissection	NED
16	Larynx	Laringectomia partialis	NED
17	Lung	Palliative RT (30-45 Gy)	DoD
18	Larynx	RT (70 Gy), Cetuximab	NED
19	Larynx	Laringectomia partialis	NED
20	Pharynx	Total pharyngectomy, neck dissection, Cetuximab	DoD
21	Neck	Chemotherapy (CDDP/5FU), Cetuximab	DoD
22	Neck	Chemotherapy (CDDP/5-FU), neck dissection, palliative RT (30-45 Gy)	DoD
23	Tracheostoma	Surgery, RT (60 Gy)	DoD
24	Tracheostoma	No therapy	DoD
25	Tracheostoma	No therapy	DoD
26	Neck	Chemotherapy (CDDP/5FU), RT (50 Gy)	DoD
27	Oral cavity	Commando procedure	DoD
Abbreviations: RT= radiotherapy; NED= no evidence of disease; DoD= died of disease			
Data is available for 27 (41,5%) of the 65 patients			
